# Dietary *Moringa oleifera* mitigates Fluconazole-Induced immunological and spleen-histological alterations in Catfish (*Clarias gariepinus*)

**DOI:** 10.1186/s12917-024-04173-x

**Published:** 2024-07-18

**Authors:** Doaa M. Basry, Salwa Mansour, Alaa El-Din H. Sayed

**Affiliations:** 1 Zoology Department, Faculty of Science, South Valley University, Qena, Egypt; 2 Zoology Department, Faculty of Science, Assiut University, Assiut, 71516 Egypt; 3 Molecular Biology Research and Studies Institute, Assiut University, Assiut, 71516 Egypt

**Keywords:** Fluconazole, *Moringa oleifera*, *Clarias gariepinus*, Immunity, Antioxidant activity

## Abstract

Fluconazole (FCZ), an antifungal from the azole family, causes several detrimental effects in fish. In recent times, there has been a notable surge in interest regarding the utilization of *Moringa oleifera* (Mo) as a dietary antioxidant. This research aimed to evaluate the potential protective effects of dietary *Moringa oleifera* (MO) against the adverse impacts of fluconazole in the African catfish (*Clarias gariepinus*). The fish were allocated into four groups as follows: a control group fed a basal diet, an FCZ - exposed (200 ng/L) fed basal diet, 1% MO fed through basal diet, and an FCZ-exposed (200 ng/L) and 1% MO fed through basal diet fed group. The results showed that FCZ exposure decreased superoxide dismutase, total antioxidant capacity, and acetylcholine esterase levels. On the other hand, FCZ exposure increased malonaldehyde and cortisol levels as compared to control (*P* < 0.05). FCZ caused immunosuppressive effects in *C. gariepinus* as revealed by lower immunity indices (lysozyme and phagocytic activity and immunoglobulin level) and increased cytokine levels (IL-6 IL-1β). Histological examination of the spleen from fish exposed to FCZ showed several splenic changes. We conclude that dietary MO supplementation has the potential to alleviate the oxidative stress, restore immune response balance, and mitigate histological damage induced by FCZ exposure, thus positioning MO as an immunostimulant in *C. gariepinus* when administered alongside FCZ.

## Introduction

Pharmaceutical products, including human and veterinary pharmaceuticals, have emered as significant pollutants due to their frequent and extensive usage [1]. Azole compounds are a group of pharmaceuticals classified into imidazole and triazole, with fluconazole being one such example The compounds, such as imidazole and triazole, were created to have antifungal properties [2]. People use fluconazole (FCZ), an antifungal from the azole family, as a medication to treat fungal infections [3]. However, it is also present in common household products including skin creams, toothpaste, soap, shampoo, and shower gels [4]. Furthermore, FCZ is widely used as a fungicide in agricultural applications and as a biocide in various products [3]. FCZ enters ecosystems from human and animal excrement, as well as waste from the pharmaceutical industry, wastewater treatment plants, clinics, hospitals, and homes. Research studies have indicated that FCZ is more hazardous in comparison to other triazole chemical compounds. However, there are few studies that investigate the harmful toxic effects this drug may have on aquatic organisms [3]. The widespread usage of azoles has resulted in enormous amounts of azole residues entering the environment, causing numerous threats to human and environmental health [5, 6]. Limited researchers that have investigated the prevalence of FCZ in South African water found 302.38 ng/L concentration of this antifungal drug in wastewater [7]. Fluconazole detected in surface water of South Africa 271.1 ng/L [6]. Few studies showed the toxic effects of fluconazole on fish at concentrations over 100 mg/L [3, 8].

Antibiotic residues are regarded as contaminants in the environment [9]. So recently, many studies have focused on using new alternative techniques which safer for the health of people, animals, and environment to minimize or replace antibiotics. One of these alternatives is medicinal plants and bacteria [10–13]. These medicinal plants are economical and have effective compounds that improve fish immunological responses and to enhance fish growth and general health [10, 14–16]. Leaf extracts from medicinal plants have been applied in several attempts to offer antioxidant protection and detoxification to fish living in contaminated environments [17, 18]. *Moringa oleifera* (*M. oleifera*) is medicinal plant which is grow fast and present a lot in the subtropics and tropics countries throughout the world with several economic importance [11]. Several studies indicate *M. oleifera* has an antioxidant, hepatoprotection, antimicrobial properties, anticarcinogenic, anti-inflammatory, and immunomodulatory properties [17, 19] because leaves of *M. oleifera* have a high concentration of elements, protein, and vitamins [9, 11, 19] with also phytochemicals like flavonoids and phenolic acids [17]. Many studies have demonstrated the antimicrobial activities of moringa leaves against pathogenic bacteria [20, 21]. Many researchers have used *M. oleifera* leaf in the diet of *Clarias gariepinus* and *Oreochromis niloticus* as a partial substitute for soybean meal and other plant proteins [9] and could additionally boost innate immune responses and strengthen defense against the challenged pathogenic infectious agents [19]. In addition, the inclusion of moringa leaf meal in a catfish diet at a rate of 100 g/kg does not have any adverse effects on feed utilization, growth, digestion of nutrients, and blood biochemistry [22]. Accordingly, this investigation aimed to examine the protective effect of *M. oleifera* against the negative impacts of fluconazole on immunity and spleen- tissue of the African catfish (*Clarias gariepinus)*.

## Materials and methods

### Fluconazole

Fluconazole (C13H12F2N6O), {Diflucan drug, 150 mg hard gelatin capsules, was obtained from a drug store. A freshly prepared stock solution of fluconazole was prepared (150 mg, one capsule in 1000 ml deionized water).

### *Moringa oleifera* preparation

*Moringa oleifera* was purchased as a dried powder from Algal Bio Co., Ltd., Japan. *Moringa oleifera* leaf contains numerous medicinal components, including potassium, calcium,, iron, phosphorus, vitamins D, and A, and flavonoids [17, 23]. When combined with commercial fish food (10 g of MO per kg of commercial fish diet), *Moringa* was prepared and mixed with fish feed. 10 g of moringa powder were weighed, dissolved with distilled water, and then combined with 1 kg of feed. It was left to dry and was used as fish food in special moringa groups.

### Fish and experimental design

Adult African catfish (*Clarias gariepinus*) *n* = 96, At a weight of 199 ± 10 g, and 28 ± 0.4 cm in length were collected from Aquatic Culture Unit at Assiut University and transferred to the Fish Biology and Environmental Pollution Laboratory at the Faculty of Science, Assiut University. The fish were acclimated for a duration of two weeks in glass aquariums with dimensions of 100 cm x 70 cm x 50 cm filled by water which had physicochemical measured characteristics (conductivity 260.8 mM/cm, pH 6.4, dissolved oxygen 6.9 mg/L, temperature 28 °C, and photoperiod 12:12 h light: dark). A random selection of fish was made for the trail and allocated to four groups (24 fish per group/ 8 in each triplicate).Fish were classified as Group1, was designated the control group (fed a basal diet without any additives), Group 2 was fed a basal diet and exposed to 200 ng/L of fluconazole, Group 3 was fed a diet containing 10% MO (10 g/kg diet) and exposed to 200 ng/L of fluconazole, and Group 4 was fed a diet just containing 10% MO (10 g/kg diet) according to Gbadamosi OK, Osungbemiro [24], and Idowu et al. [25]. Throughout the trial’s period, the MO-nonfeeding fish were supplied with commercial food only (Skritting company, Egypt) once a day, about 3% of their body weight, and the water was changed every day (40%) to eliminate the impact of fish waste and to re-dose. After a 15-day exposure, six fish were randomly sampled from each group (two from each replicate). Fish were anesthetized using 200 ppm solution of clove powder [26]. After cutting the tail and collecting blood from the caudal vein, blood was placed into non-heparinized tubes for antioxidant and immunological parameters.

### Antioxidant parameters

Superoxide dismutase (SOD) was assessed based on its ability to inhibit the phenazine methosulphate-mediated reduction of nitroblue tetrazolium dye to form a red product [27]. Utilizing kits, total antioxidant capacity (TAC) was determined (Sigma-Aldrich, USA), Evaluation was based on the colorimetrical determination of hydrogen peroxide (H2O2) that remained following the reaction of antioxidants in the specimen with a defined amount of exogenously provided H2O2 [28]. Malondialdehyde (MDA) was measured using a thiobarbituric acid reaction and tetramethoxypropane as an external standard [29]. Stanbio kits were used to assay serum acetylcholinesterase (AchE) by the procedure mentioned by Knedel and Böttger [30]. According to Foster and Dunn [31], The levels of cortisol were assessed using ELISA kits (Human Ultrasensitive, Biosource International Inc.).

### Immunological parameters

. Individual serum samples were obtained from the blood samples using centrifugation at a speed of 5000 rpm per minute for duration of 10 min. These samples were stored for the purpose of estimating immunological indices. Lysozyme (LYZ) activity was measured using a turbidity assay technique [32]. Immunoglobulin M (IgM) levels in the serum samples were quantified using an ELISA kit according to a previously described method [33]. The phagocytic (PA) activity (%) of leukocytes was determined using a formula described elsewhere [34]. The levels of Interleukin-β and − 6 (IL-1β and − 6) were measured using the methods indicated in the references [35, 36] by ELISA kits (Human Ultrasensitive, Biosource International Inc.).

### Spleen histopathological examination

Following a period of 15 days, four fish were manipulated from each group (control and treated); spleen was obtained from each fish and subsequently washed with neutral saline. Subsequently, every tissue sample was preserved in neutral-buffered formaldehyde, dried with ethanol, cleared in methyl benzoate, embedded in wax, and then sectioned into 5 µ-thick Sect. [37]. After dewaxed and rehydrated slides underwent hematoxylin and eosin (H&E) staining [38]. Stained sections were examined using a VE-T2 microscope, and selected regions were photographed using a 14MP OMAX camera (MN: A35140U3, China).

### Statistical analysis

The mean and standard error of the mean values were determined. Statistical differences between the test groups were analyzed by one-way analysis of variance in SPSS 45 at the 0.05 significance level (*P* < 0.05). Post hoc comparison was done using Tukey’s-b and Dunnett tests (SPSS V.26).

## Results

### Antioxidant parameters

Exposing *C. gariepinus* to FCZ revealed a highly significant (*P* < 0.0001) decline in the levels of SOD and TAC compared with the control group while MO group didn’t show any significant changes for both parameters. *C. gariepinus* were fed a combination of FCZ and MO showed a significant (*P* < 0.05) decrease in SOD to levels near to control. Lipid peroxidation displayed a highly significant (*P* < 0.0001) increase in the FCZ exposed group, while a decrease in fish fed with MO alone (Group 3) or combined with FCZ (Group 4) to control values (Table 1). *C. gariepinus* exposed to FCZ displayed a significant decline in acetylcholinesterase (AchE) and a significant rise in cortisol levels compared to fish in the control group. The combination of FCZ with MO for 15 days exhibited improvement towards control levels (Table 1).

### Immunological parameters

*C. gariepinus* exposed to FCZ for 15 days exhibited a highly significant (*P* < 0.0001) decrease in measured immunological components such as lysozyme (LYZ) & phagocytic activity (PA), and immunoglobulin M (IgM) levels, compared to control. Dietary supplementation with MO in the FCZ + MO group of fish reduced the negative effects of FCZ and ameliorated the previously measured biomarkers, but it didn’t reach the control values (Table 2).

*C. gariepinus* exposed to FCZ for 15 days displayed a highly significant (*P* < 0.0001) increase in the secretion of interleukins (IL-1β and IL-6) compared to those in the control group. The secretion levels of (IL-1β and IL-6) decreased by adding MO in the FCZ + MO group and improved the immunological parameters near to control levels (Table 2).

### Histopathological changes of spleen

**Fig. 1:**
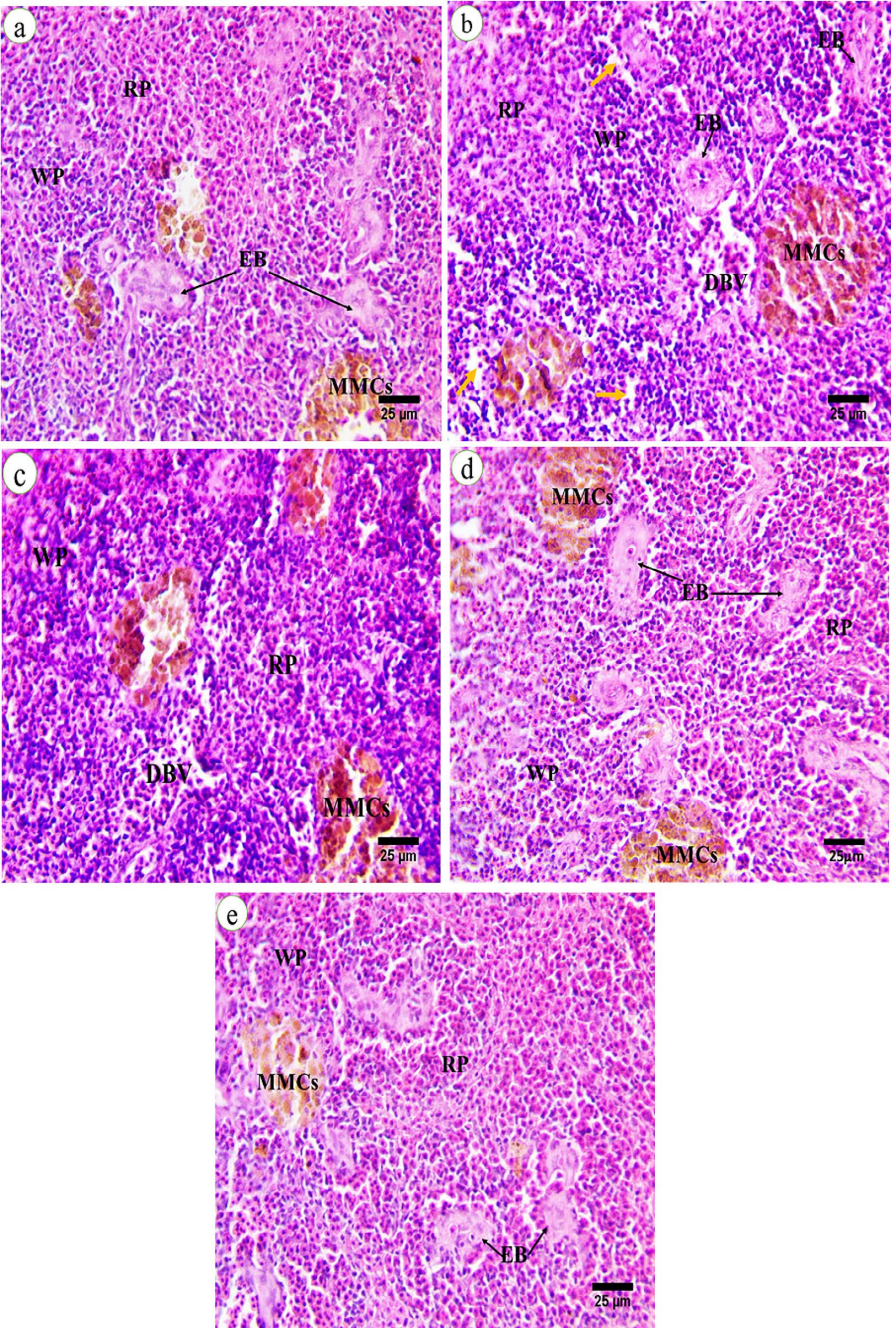
**(a-e)**, Spleen sections from *Clarias gariepinus* of all experimental groups stained by (H&E) illustrating the normal morphological structure of fish spleen in the control group (**a**). Selective deformations of shape following FCZ treatment at 200 ng/L (**b, c**) for 15 days, fish exposed to fluconazole (200 ng/l) + 10% Moringa (10 g/kg) for 15 days (**d**). Fish supplemented with 10% Moringa (10 g/kg) for 15 days (**e**). Red pulp (RP), white pulp (WP), Ellipsoid bodies (EB), Melanomacrophage centers (MMCs), Red blood corpuscles (RBC´s), dilated of blood vessels (DBV), the spleen parenchyma slightly loose (yellow arrows). (X- 40, Scale Barr = 25 μm)

Spleen section of control *C. gariepinus* showed that the that the normal architecture of the spleen contained white pulp (WP) consisting of aggregated lymphoid cells, a red pulp (RP) containing mainly red blood corpuscles, an interconnecting system of splenic cords that contains in their core ellipsoid bodies (EB) surrounded by foci of lymphocytes, and a few large melanomacrophage centers (MMCs) with macrophage (pigmented) cells (Fig. 1a). Catfish after exposure to FCZ (200 ng/L) for 15 days showed alterations in splenic tissue, including the spleen parenchyma, which is slightly loose (yellow arrow), the unclear boundary of two pulp (W&R) pulps, the expansion white pulp with large numbers of lymphocytes, and the shrunken red pulp with few numbers of red blood cells due to the increase in lymphocyte production inside the white and red pulps. Diffuse activation of melanomacrophage centers (MMCs) that increased in number and appeared deep in color compared to the control group, dilation of blood vessels (DBV), which were full of red blood corpuscles (RBC’s) and shrunken of ellipsoid bodies (EB) were also observed when compared with the control group (Fig. 1b, c). Fish exposed to 200 ng/L fluconazole and 10% Moringa for 15 days showed improvement in the morphology of splenic tissue with few histological changes. Whereas showed unclear boundary of two pulp (W&R) pulps due to few dispersions of lymphocytes in both white and red pulps, increment of both Melano macrophage centers, and dilation of ellipsoid bodies when observed with different shapes compared with both the control and FCZ groups (Fig. 1d). While groups supplemented with Moringa (10 g/kg) for 15 days showed amelioration in splenic morphology more or less similar to control groups, clear boundaries of W&R pulps were observed, and lymphocytes aggregated in white pulp, which surrounded the ellipsoid structures. Clear red pulp full of red blood corpuscles and aggregation of melanomacrophage centers were observed in (Fig. 1e).

**Table 1:** The effects of fluconazole and *M. oleifera* on antioxidant parameters of African catfish *(C. gariepinus)* after 15 days of exposure

Treatment	Control	FCZ	MO	FCZ + Mo
SOD (U/ml)	2.70 ± 0.06^c^	1.97 ± 0.07^a^	2.82 ± 0.06^c^	2.29 ± 0.11^b^
TAC (nmol/L)	54.89 ± 3.28^b^	43.34 ± 0.95^a^	53.77 ± 0.76^b^	44.12 ± 0.33^a^
MDA (nmol/ml)	15.76 ± 1.09^a^	30.09 ± 2.26^b^	17.08 ± 0.72^a^	18.12 ± 0.29^a^
Acetylcholinesterase (AchE) (µ/L)	551.65 ± 4.53^c^	434.24 ± 6.78^a^	564.92 ± 3.91^c^	533.24 ± 4.84^b^
Cortisol (µg/dL)	13.72 ± 0.60^ab^	16.12 ± 0.51^c^	14.72 ± 0.39^bc^	12.44 ± 0.08^a^

Data are presented as the mean ± standard error (*n =* 6). Row with different superscript letters indicated significant difference between groups (*P* < 0.05)

**Table 2:** The effects of fluconazole and *M. oleifera* on immunological parameters of African catfish *(C. gariepinus)* after 15 days of exposure

Treatment	Control	FCZ	MO		FCZ + MO
Lysozyme activity (LYZ) (mg/mL)	14.34 ± 0.18^c^	8.47 ± 0.40^a^	13.45 ± 0.25^c^		10.56 ± 0.16^b^
Immunoglobulin (IgM) (µg/mL)	34.99 ± 0.26^d^	24.88 ± 0.34^a^	33.7 ± 0.27^c^		28.997 ± 0.41^b^
Phagocytic activity (PA) (%)	24.14 ± 0.24^d^	16.64 ± 0.31^a^	22.08 ± 0.22^c^		19.57 ± 0.18^b^
Interleukin-1 beta (IL-1β) (pg/mL)	10.73 ± 0.08^a^	18.51 ± 0.47^d^	13 ± 0.34^b^		15.76 ± 0.22^c^
Interleukin (IL-6) (pg/mL)	51.77 ± 0.17^a^	61.46 ± 0.19^d^	53.47 ± 0.18^b^		56.09 ± 0.36^c^

Data are presented as the mean ± standard error (*n =* 6). Row with different superscript letters indicated significant difference between groups (*P* < 0.05)

## Discussion

Azole compounds have negative impacts on fish, as demonstrated by variations in biomarkers of oxidative stress and neurotoxicity, according to reports [39]. SOD activity as well as TAC level strong indicators for exposure to reactive oxygen species (ROS) [40].

In the present study, the negative impacts of fluconazole on catfish *(C. gariepinus)* could be represented by induction of the oxidative stress, our results showed that exposure to FCZ reduced both SOD activity and TAC level while increased malondialdehyde levels in comparison with normal fish. The same trends were reported by other researchers after exposure to FCZ in zebrafish embryos [41] and in *Gobiocypris rarus* embryos [42] who suggested that inhibition in SOD activity at high doses could be attributed to destruction in the protective system of fish embryos or as a result of triazole effects such as weakness of antioxidant defense system which increases the oxidative stress and leads to damage of some physiological functions of *G. rarus* embryos. SOD is an antioxidant enzyme that serves as the initial line of defense and protection for the body against undesirable oxidative stress by converting both superoxide anion and toxic reactive oxygen species into hydrogen peroxide [43], which may lead to the oxidation of cysteine which cause inhibition in SOD activity [44].

Lipid peroxidation occurs by the combination of ROS with the cellular membrane components due to the impairment of permeability of the cell membrane [45]. The presence of access oxidative stress is accompanied by an increase in malondialdehyde level (MDA) [46], which causes cellular membrane damage and cellular function modification due to antioxidant enzymes lack [17]. The production of significant amounts of free radicals as induced by oxidative stress is the main reason for MDA production [47]. Our findings were consistent with Li et al., [48] who informed us of a significant decrease in SOD enzyme and raise in MDA in *O. mykiss*, in Medaka fish *O. latipes*, and in zebrafish subjected to propiconazole and difenoconazole. Atama [49] reported a significant decrease in SOD enzyme in catfish subjected to the antipsychotic drugs chlorpromazine. This inhibition in enzyme activity may result from more production of (ROS) due to azole [50]. Any defect in the regulation of SOD activity has been associated with the presence of diseases or other cellular disorders [51]. Oruç and Usta [52] reported that activation or inhibition of antioxidant enzymes depends on a variety of elements, including the species of fish, its sensitivity, and the exposure period to the stressor. The antifungal activity of different types of azoles is affected by oxidative eruption. So, information on changes in the oxidative stress indices is essential to evaluating the risk of azole pollution [53]. Also, exposition to various pharmaceuticals induced a significant elevation in LPO in the gill and liver tissues of *C. gariepinus* [54]. The same results were observed by Sehonova et al., [55] after exposure of zebra fish larvae to naproxen and explained as an indicator of exposure to oxidative stress. The recorded oxidative stress and changes in SOD, TAC, and MDA after exposure to FCZ were supported by other studies in *C. gariepinus* treated with metformin [56]. The observed histological alterations in some fish organs may be linked with the oxidative damage that occurred in these tissues which cause dysregulation of SOD activity and elevation in MDA [57], as explained in this research.

In this research, moringa was used to investigate the extent of its mitigation and ameliorative role against negative impacts caused by FCZ in catfish. Our result showed that the addition of dietary MO in the (FCZ + MO) group could diminish the negative impacts of FCZ on catfish. *M. oleifera* Contains polyphenols components such as flavonoids, phenyl propanoids, tannins, and phenolic acids which donate or accept electrons to stabilize the induced free radicals from cells (hydrogen donors) enhancement the antioxidant effect of MO which helps *C. gariepinus* to improve their antioxidant response [58]. Various studies have demonstrated that diet supplementation with MO could improve fish antioxidant response against a variety of toxicants in Nile tilapia including fipronil [19], pendimethalin [23], sodium fluoride [59]. The same results were recorded by Abd El-Gawad et al., [60] in Nile tilapia fed by MO-supplemented diets against bacterial infection by *Aeromonas hydrophila*. The antioxidant characteristics of *M. oleifera* act as scavengers that prevent and inhibit free radical formation and break down peroxidases [61]. In addition, *M. oleifera* leaves are rich in vitamin C, organic acids, and folic acids that possess antioxidant properties [62].

The AChE enzyme plays a significant role in neurotransmission and serves as a valuable biomarker for evaluating neurotoxicity [53]. It acts as the main reason for the hydrolytic dissolution of the neurotransmitter acetylcholine into the inactive outputs of choline and acetic acid. The role of AChE in cholinergic transmission process is to control and regulate the nervous synaptic transmission by decreasing the amount of acetylcholine in the junctions [63]. Exposure to different toxicants causes inhibition in AChE activity which increases the neural junctions causing continuous nerve fiber or muscle stimulation causing many dangerous symptoms of respiratory failure, paralysis, and death [64]. According to our findings, *C. gariepinus* treated with FCZ showed lower AChE levels in compared to control. The inhibition in AChE levels may result from the direct action between the FCZ and certain active sites in the enzyme working to hydrolysis of acetylcholine [65]. Also, other studies are compatible with our results as in *G. rarus* when exposed to fluconazole [42], In *C. auratus*, treatment with 0.2–20 mg/L of ketoconazole [39], in zebrafish larvae after exposure to imidazole [66]. Studies have demonstrated that the effects of environmental compounds, such as pharmaceuticals, in aquatic environments inhibit AChE concentration [67]. Taher et al., [56] explained that the inhibition of AChE level by increasing the drug concentration may be part of cell protection against cellular oxidative stress induced by the drug. Furthermore, [68] suggested that a decrease in AChE levels could affect the ability of fish to feed.

Cortisol is a strong indicator that shows exposure to stress which, cortisol levels increase by increasing stressors [69]. Our result showed that Cortisol levels were highly significantly increased by FCZ exposure. This can be attributed to the correlation between FCZ action and the increase of hormonal secretion in the pituitary gland, as it normally releases adrenocorticotropin hormone, which enhances the adrenal glands to produce cortisol hormone in the blood. The findings we obtained were likewise documented by Khalil et al., [70] who observed an increase in Adrenocorticotropic hormone (ACTH) in Albino Rats after treatment with Ketoconazole. Bisson and Hontela [71] documented the same outcomes in *O. mykiss* and, in catfish *(C. gariepinus)* after exposure to mancozeb-fungicide and monocyclic aromatic hydrocarbons, respectively. Gesto et al., [72] proposed that continuous exposure to various contaminants may reduce the sensitivity of ACTH or even non-regulation of its level which reduces the cortisol hormone response to a certain stressor. Additionally, [73] supposed that exposure to stressors for a long period may increase corticosteroid elimination as time increases.

In our study, the improvement in AChE activity and cortisol levels in the FCZ-exposed groups that were fed meals containing *M. oleifera* may be attributed to *M. oleifera* leaves containing antioxidant components, such as vitamin C and E, which can restore AChE properties and mitigate the oxidative stress induced after toxicant exposure [74]. These outcomes aligned with Ibrahim et al., [75] who recorded that in *O.niloticus* significant restoration of AChE activity was reported in chlorpyrifos (CPF) treated fish fed the basal diet containing 10% *M. oleifera* leaves for 15 or 30 days compared to CPF-treated fish. Also, restoration in cortisol levels was reported by Hamed and El-Sayed [23] after treatment with MO (20 ml/ 30 l water) against pendimethalin.

The enzyme LYZ, which is secreted by fish white blood cells and acts as an antibacterial, contributes to preventing bacterial infection, and that considered one of the most active non-specific immune response tools [76]. One of the principal immunoglobulins is immunoglobulin M (IgM) that is supposed to have the most sufficient immunological response among the components of the humoral immunity of fish [77]. Phagocytic activity plays an important role in the defense of fish against pathogenic microorganisms [78]. Various toxicants cause alterations in humoral immunity and cellular immunity, that can have negative effects on fish [75]. In this study, the activity of immunological parameters (LYZ), (IgM), and (PA) levels in catfish after exposure to 200 ng/L of FCZ showed a highly significant decrease, and in contrast, the interleukins IL-1β and IL-6 showed a highly significant increase compared to the control group. This is aligned with Saha et al., [79] who reported a lower lysozyme activity in *Labeo rohita* fed with FCZ at ≥ 20 mg kg BW^− 1^ on the 30th day of feeding. The decline in (LYZ), (IgM), and (PA) levels and increase in interleukin (IL-1β and IL-6) levels were reported in *Cyprinus carpio* and in *C.gariepinus* that was administered difenoconazole and pyrogallol [80], in *O. niloticus* treated with Abamectin [58]. According to Mohamed et al. [81] a reduction in phagocytic activity leads to a decline in the LYZ level. Although the study conducted by Saha et al. [79] illustrated the FCZ role in the enhancement of fish immunity against infection when supplemented at a dose of 10 mg kg BW^− 1^ it didn’t explain LYZ inhibition when the dose at ≥ 20 mg kg BW^− 1^.In fish, cytokines poses a network of defense in the immune system [82] that produce proteins that can control and regulate the immune responses [83]. IL-1β has the ability to activate pro-inflammatory cytokines such TNF-α, IL-6, IL-8, and IL-18 [84]. A similar elevation in both interleukin (IL-6 and IL-1β) levels was demonstrated by Zhao et al., [83] after treatment of zebrafish with 0.5-mg/L propiconazole. Taher et al., [56] reported that cytotoxic activities may lead to cell apoptosis. This elevation may be linked to increase ROS which induces inflammation and cell damage as a result of FCZ action.

The current research indicated the palliative effect of MO against FCZ immunotoxicity as improvement in immunological parameters were recorded in the FCZ + MO group compared with the FCZ group. Such improvement has been proven in several studies using MO in protection against toxicants, abamectin [58], chlorpyrifos [75]. This modulation in immune function may result from MO role in modifications of cellular membranes as it is rich in fatty acids (e.g. oleic acid, palmitic, and stearic acid) [85].Additionally, containing MO compounds such as vitamins (C, A, K) and amino acids that improve immunoglobulin formation that promote immunity [86].

In bony fish, the spleen is the principal lymphoid organ and serves as an immune store by containing lymphocytes [87]. The mature lymphocytes and the development of the active immune response in fish primarily occur in the white pulp [88]. Increased lymphocytes production inside white and red pulps as in our study was also recorded in *O. niloticus* after treating with florfenicol [89], in *C. gariepinus* after exposed to fluoride for 60 days [88]. Cytologically Sites of tissue injury frequently exhibit an accumulation of melanomacrophage centers (MMCs) [90]. Moreover, splenic tissues exposed to FCZ showed diffuse activation of melanomacrophage centers that increased in number and appeared deep in color. These results are in line with the results of *C. gariepinus* after exposure to various toxicants [71]. An increase in MMCs was associated with histopathological alterations, suggesting that oxidative stress induced lymphocyte aggregation, indicates an immune response [91]. Herraez and Zapata [92] suggested that a decline in lysozyme activity in fish was linked to MMCs.

In comparison to FCZ exposure, the inclusion of MO in fish diets showed marked improvement in morphology of splenic tissues. While fish supplemented with *M. oleifera* only (10 g/kg) for 15 days showed slight amelioration in splenic morphology compared to control. This result suggests that moringa may provide protection against the negative impacts of FCZ. These results are incompatible with Abdelhiee et al., [93] reported that the spleen of Nile tilapia-fed diet supplemented with 0.5% *M. oleifera* extract exhibiting a spleen structure that is virtually normal, thereby ameliorating the splenic changes caused by aflatoxin B1 toxicity.

## Conclusion

The present study investigated the potential protective effects of MO in fluconazole (FCZ)- exposed *C. gariepinus*. Exposure of *C. gariepinus* to FCZ results in oxidative stress, serum AChE suppression and elevation of serum cortisol levels. FCZ, also, have negative impacts on immunological and anti-inflammatory responses including damage to splenic tissue in C. *gariepinus*. *Moringa olifera* (MO) leaf extract, aa natural herbal phyto-additive supplement, was found to enhance the overall health by protecting fish against the negative impacts of FCZ. Since studies on the negative effects of azole antifungal are limited in fish, additional studies are needed to assess impact of continuous exposure to high concentrations of these antifungal agents.

## Data Availability

No datasets were generated or analysed during the current study.
